# Hypertriglyceridemia Induced Pancreatitis in a Non-Diabetic Pregnant Patient Requiring the Use of Total Parenteral Nutrition

**DOI:** 10.4021/gr299w

**Published:** 2011-03-20

**Authors:** Ariyo Ihimoyan, Haritha Chelimilla, Nirisha Kalakada, Anil Dev, Kavitha Kumbum

**Affiliations:** a Division of Gastroenterology, Bronx Lebanon Hospital Center, Bronx, New York 10457, USA; b Department of Medicine, Bronx Lebanon Hospital Center, Bronx, New York 10457, USA

**Keywords:** Total parenteral nutrition, Gestastional hypertriglyceridemic pancreatitis

## Abstract

Hypertriglyceridemia induced pancreatitis in pregnancy is established and has been widely reported. However there are very scanty reports of cases involving the use of total parenteral nutrition. We report the case of a 37-year-old gravida 3, para 2 woman at 34 weeks of gestation who presented with one day of severe epigastric pain radiating to the back, nausea and bilious vomiting caused by pancreatitis induced by hypertriglyceridemia. Her initial serum triglyceride, amylase and lipase concentration were 6,552 mg/dl, 314 U/L and 537 U/L respectively. She initially received intravenous fluids and insulin with reduction of serum triglyceride levels to 583 mg/dl on the fifth day of admission. However attempts to refeed the patient with solid food resulted in induction of hypertriglyceridemia and relapse of pancreatitis. Lipid free total parenteral nutrition was commenced in the third week of admission and lead to a better control of triglyciderides and resolution of pancreatitis until delivery of a full term healthy neonate.

## Introduction

Acute pancreatitis is an uncommon complication in pregnancy occurring in approximately 3 in 100,000 pregnancies [1]. It is often caused by biliary diseases [2]. Hypertriglyceridemia induced pancreatitis in pregnancy is a serious complication that is associated with a significant risk of death for both mothers (21%) and fetuses (20%) [3]. Serum triglyceride concentrations increase during normal pregnancy and reach maximal levels in the third trimester of pregnancy [4]. This is due to a combination of a human placental lactogen-related increase in adipose tissue lipolysis and hepatic synthesis of very low density lipoprotein which result in increased production of triglyceride-rich lipoproteins [5]. The physiological increase in triglyceride levels that occurs during pregnancy does not reach the threshold of 1,000 mg/dl that would induce pancreatitis [6]. However severe hypertriglyceridemia may result if the normal increased production of triglyceride-rich lipoproteins occurs in conjunction with genetic disorders in triglyceride metabolism. This marked hypertriglyceridemia may lead to pancreatitis, hepatomegaly and eruptive xanthomas [7, 8]. The most common disorders of triglyceride metabolism are familial hypertriglyceridemia, lipoprotein lipase deficiency, and apolipoprotein C-II deficiency.

Management of acute pancreatitis in non-pregnant patients includes generous intravenous hydration, pain control and no oral intake to ensure adequate bowel rest. These measures may be effective in controlling the triglyceride levels in pregnant patients but their duration is very limited because a growing fetus requires maternal calories, essential amino acids and essential fatty acids. However early initiation of oral feeding in these patients may exacerbate pancreatitis and also lead to worsening triglyceride levels. Minimal fat oral diet contains a high concentration of non-fat calories and its use in pregnancy may lead to increased synthesis of very low density lipoproteins which leads to enhanced production of triglycerides by the liver [9]. Total parenteral nutrition is an effective means of providing the necessary calories and essential amino acids for the growing fetus while controlling the maternal triglyceride concentrations and preventing the induction of pancreatitis. We herein report a case of severe hypertriglyceridemic pancreatitis in a pregnant patient and the successful use of total parenteral nutrition in the management of the patient.

## Case Report

A 37-year-old gravida 3 para 2 woman at 34 weeks of gestation presented with severe epigastric pain radiating to the back of one day duration associated with nausea and bilious vomiting. There was no medical history of gestational diabetes or gallstone disease. She denied alcohol abuse, drug intake or oral contraceptive usage. Her prior deliveries were uneventful. Family history was significant for pancreatic cancer in her father. Her home medications included prenatal vitamins and iron supplementation. On admission, the patient’s blood pressure was 113/65 mm Hg, her pulse was 120/min and respiratory rate was 30/min, her temperature was 37.0 °C. Fetal heart rate was between 130 to 150/min. There was no evidence of eruptive xanthomas. Her abdominal examination revealed a gravid uterus and epigastric tenderness. Laboratory findings include serum levels of amylase, lipase and triglyceride levels of 314 U/L (normal: 16 to 100), 547 U/L (normal: < 61) and 6,552 mg/dl (normal: 58 to 150) respectively. This was consistent with a diagnosis of acute pancreatitis due to hypertriglyceridemia. Her basic metabolic profile, liver function tests were within normal limits. An abdominal ultrasound revealed an unremarkable gallbladder, fatty liver and the pancreas could not be adequately visualized.

**Figure 1:**
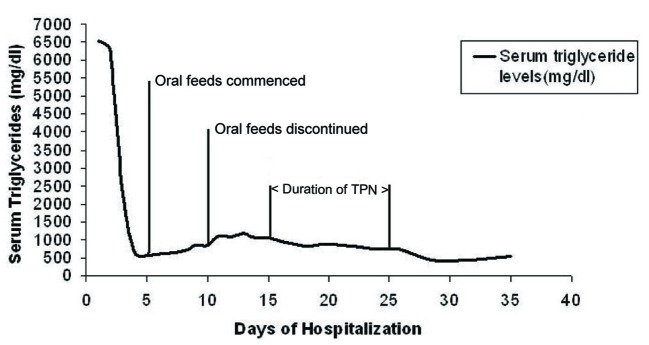
Trend of triglyceride levels during the course of hospitalization. TPN: total parenteral nutrition.

She was initially given nothing by mouth and started on intravenous insulin drip along with 5% dextrose. After five days the triglyceride level decreased to 583 mg/dl and her symptoms had resolved. Oral feeds were commenced on the same day. There was a rebound increase in triglycerides over the next four days to levels up to 1,040 mg/dl. She also developed recurrence of her symptoms which necessitated resumption of nothing by mouth. Due to concern for the nutritional status of mother and fetus, total parenteral nutrition was initiated on day 16. The total parenteral nutrition was lipid free and comprised of 60 gm/l of amino acids, 600 kcal/l of dextrose, sodium chloride at 60 meq/day, potassium acetate at 20 meq/day, calcium gluconate at 4.5 meq/day, magnesium sulfate at 6 meq/day, multivitamins and trace elements. She received total parenteral nutrition for a total of ten days after which an oral low fat diet was resumed. Three days later intravenous insulin was discontinued. From the day of commencement of total parenteral nutrition until discharge, her triglyceride levels remained controlled (range between 430 - 1,014 mg/dl) and there was no recurrence of pancreatitis. The trend of triglyceride levels during the course of hospitalization is shown in Figure 1. She was eventually delivered of a healthy neonate by cesarean section. The patient was discharged in a stable condition with a triglyceride level of 540 mg/dl on niacin, lovaza (omega-3 fatty acid) and gemfibrozil.

## Discussion

The goals of management of pregnant patients with hypertriglyceridemia induced pancreatitis should include decreasing the serum triglyceride concentration and pancreatic activity while supplying maternal and fetal nutritional needs. Achieving these goals may be quite challenging. Therapies like plasma exchange, use of gemfibrozil and extracorporeal lipid elimination may be effective in controlling triglyceridemia but do not meet the nutritional requirements of mother and child [10, 11].

Avoidance of oral diet and intravenous administration of 5% dextrose along with insulin often lead to a dramatic reduction of serum triglycerides [12]. However our patient’s triglyceride levels increased upon her initial refeeding trial. Intravenous 5% dextrose does not supply enough calories and could not be used for the extended duration required for enteric rest.

Dietary modification with a minimal fat oral diet should theoretically reduce chylomicron levels and decrease serum triglyceride concentration. However it may paradoxically increase the serum triglyceride levels by inducing production of triglyceride rich lipoprotein. The growing fetus requires essential fatty acids and amino acids for development and maturity of vital organs like brain and lung. Parenteral nutrition provides a viable means for the fetus to receive these supplements.

Total parenteral nutrition with up to10 percent of calories as fat does not significantly increase maternal serum triglycerides. This is because of the systemic delivery of lipids which bypasses the liver where production of triglyceride rich lipoproteins occurs. It also enables the supply of other nutritional supplements that are required by the fetus.

Total parenteral nutrition has also been used as an effective alternative to surgical treatment of chronic cholecystitis in the second and third trimester [13]. A study of ten pregnant patients with severe hyperemesis managed with parenteral nutrition found no adverse effect on maternal weight gain and fetal growth [14].

The first reported case of total parenteral nutrition use in gestational hyperlipidemic pancreatitis was described by Weinberg et al [9]. The patient’s symptoms and triglyceride levels were only controlled after initiation of lipid free total parenteral nutrition; however the fetus developed intrauterine fetal growth retardation. Our patient was delivered of a healthy neonate with no compromise on fetal growth.

Parenteral nutrition in pregnancy should be managed preferably by an experienced clinical nutrition support staff. The major complications are related to central venous catheter placement and include pneumothorax, hemorrhage and rarely death [15]. There has been recent trend towards use of peripherally inserted central catheters (PICC) because of lower rate of major complications and relative ease of insertion compared to central venous catheters [15]. PICCs should always be considered particularly in high risk populations like pregnant women. Several studies however have shown a higher rate of minor complications like thrombophlebitis in patients with PICCs [16, 17]. PICC insertion is highly operator dependent and lowest complication rates have been seen in the most experienced centers [18].

The clinical course of pancreatitis during pregnancy may be complicated by pulmonary and circulatory collapse, sepsis, Adult Respiratory Distress Syndrome and pseudocysts. Perinatal mortality and morbidity in pregnant patients with acute pancreatitis is associated with high rate of preterm birth (about 60%) [19] and the estimated perinatal mortality is between 10 - 32% [20]. However, out of 14 cases of gestational hyperlipidemic pancreatitis, no maternal death was reported [20].

In conclusion, hypertriglyceridemia induced pancreatitis is a serious complication of pregnancy that can occur in patients with familial hypertriglyceridemia. It necessitates prompt control of serum triglycerides while maintaining maternal and fetal nutritional support. Total parenteral nutrition serves as an effective means of controlling symptoms and serum triglycerides when early oral feeding leads to recurrent symptoms and poor control of serum triglyceride concentration. It also optimizes nutritional delivery to the growing fetus.
